# A Switch in Hepatic Cortisol Metabolism across the Spectrum of Non Alcoholic Fatty Liver Disease

**DOI:** 10.1371/journal.pone.0029531

**Published:** 2012-02-20

**Authors:** Adeeba Ahmed, Elizabeth Rabbitt, Theresa Brady, Claire Brown, Peter Guest, Iwona J. Bujalska, Craig Doig, Philip N. Newsome, Stefan Hubscher, Elwyn Elias, David H. Adams, Jeremy W. Tomlinson, Paul M. Stewart

**Affiliations:** 1 Centre for Endocrinology, Diabetes and Metabolism, Institute of Biomedical Research, School of Clinical and Experimental Medicine, University of Birmingham, Birmingham, United Kingdom; 2 Wellcome Trust Clinical Research Facility, University Hospital Birmingham NHS Foundation Trust, Birmingham, United Kingdom; 3 Centre for Liver Research and NIHR Biomedical Research Unit in Liver Disease, University Hospital Birmingham NHS Foundation Trust and University of Birmingham, Birmingham, United Kingdom; 4 Radiology, University Hospital Birmingham NHS Foundation, Trust, Birmingham, United Kingdom; 5 Pathology, University Hospital Birmingham NHS Foundation Trust and University of Birmingham, Birmingham, United Kingdom; Clermont Université, France

## Abstract

**Context:**

Non alcoholic fatty liver disease (NAFLD) is the hepatic manifestation of the metabolic syndrome. NAFLD represents a spectrum of liver disease ranging from reversible hepatic steatosis, to non alcoholic steato-hepatitis (NASH) and cirrhosis. The potential role of glucocorticoids (GC) in the pathogenesis of NAFLD is highlighted in patients with GC excess, Cushing's syndrome, who develop central adiposity, insulin resistance and in 20% of cases, NAFLD. Although in most cases of NAFLD, circulating cortisol levels are normal, hepatic cortisol availability is controlled by enzymes that regenerate cortisol (F) from inactive cortisone (E) (11β-hydroxysteroid dehydrogenase type 1, 11β-HSD1), or inactivate cortisol through A-ring metabolism (5α- and 5β-reductase, 5αR and 5βR).

**Objective and Methods:**

In vitro studies defined 11β-HSD1 expression in normal and NASH liver samples. We then characterised hepatic cortisol metabolism in 16 patients with histologically proven NAFLD compared to 32 obese controls using gas chromatographic analysis of 24 hour urine collection and plasma cortisol generation profile following oral cortisone.

**Results:**

In patients with steatosis 5αR activity was increased, with a decrease in hepatic 11β-HSD1 activity. Total cortisol metabolites were increased in this group consistent with increased GC production rate. In contrast, in patients with NASH, 11β-HSD1 activity was increased both in comparison to patients with steatosis, and controls. Endorsing these findings, 11β-HSD1 mRNA and immunostaining was markedly increased in NASH patients in peri septal hepatocytes and within CD68 positive macrophages within inflamed cirrhotic septa.

**Conclusion:**

Patients with hepatic steatosis have increased clearance and decreased hepatic regeneration of cortisol and we propose that this may represent a protective mechanism to decrease local GC availability to preserve hepatic metabolic phenotype. With progression to NASH, increased 11β-HSD1 activity and consequent cortisol regeneration may serve to limit hepatic inflammation.

## Introduction

Non-Alcoholic Fatty Liver Disease (NAFLD) is the hepatic manifestation of the metabolic syndrome and is now acknowledged to be the commonest liver problem of the western world, and the leading cause of cryptogenic cirrhosis. NAFLD represents a spectrum of liver disease ranging from simple and reversible hepatic steatosis, to non alcoholic steato-hepatitis (NASH) where there is evidence of inflammation culminating in cirrhosis with liver failure, and hepatocellular carcinoma. It is rapidly becoming the leading indication for liver transplantation. Critically, the histological diagnosis at presentation predicts prognosis in these patients. Those with simple fatty liver at presentation only have a 2% risk of progressing to end stage cirrhosis in a 20 year period. However when there is evidence of steatohepatitis or fibrosis, the risk of developing cirrhosis is up to 50% in a 2 year period [1]. The pathogenesis of NAFLD is poorly understood but several factors are thought to be important, including insulin resistance, obesity and type 2 diabetes; 90% of patients with NAFLD cirrhosis having obesity and/or diabetes mellitus.

Patients with the metabolic syndrome share many phenotypic features with Cushing's syndrome (e.g. hypertension, abdominal obesity, insulin resistance and impaired glucose tolerance). Indeed, 20% of patients with Cushing's syndrome have NAFLD [2], and there are a number of reports that implicate pharmacological glucocorticoid (GC) excess in hepatic triglyceride accumulation [3], [4]. Glucocorticoids promote steatosis by directly stimulating hepatic de novo lipogenesis and free fatty acid (FFA) utilization [5]–[7], and by promoting lipolysis within omental fat, resulting in increased portal FFA delivery to the liver [8]. Cushing's syndrome is rare and the vast majority of patients with NAFLD have normal circulating cortisol levels. However, local GC concentrations within key metabolic target tissues are controlled at the pre-receptor level through a series of enzymes; 11β-hydroxysteroid dehydrogenase type 1 (11β-HSD1), interconverting hormonally inactive cortisone (E) to active cortisol (F) and, 5α and 5β reductases (5αR and 5βR) which inactivate cortisol to the dihydro and subsequently tetrahydro metabolites (THF or 5αTHF). Our previous work has shown that in simple obesity, there is a reduction in the generation of serum cortisol from dexamethasone-suppressed values after the administration of oral cortisone reflecting decreased hepatic 11β-HSD1 activity [9]. This comes at a time of interest in the concept of selective 11β-HSD1 inhibition as a novel therapy for patients with the metabolic syndrome – inhibition of hepatic and adipose cortisol regeneration resulting in reduced gluconeogenesis and adipogenesis respectively [10]–[12].

A number of cross sectional studies have reported the association of NAFLD with chronic, subclinical general activation of the hypothalamo-pituitary-adrenal (HPA) axis in humans [13]–[15]. None of these studies however have undertaken a detailed analysis of hepatic pre receptor cortisol metabolism in patients with NAFLD. We propose that dysregulation of hepatic GC metabolism may be critical in the pathogenesis and/or progression of NAFLD with increased regeneration (11β-HSD1) or decreased clearance (5α-reductase) contributing to the hepatic phenotype. We have therefore performed a detailed characterisation (*in vivo* and *ex vivo*) of GC metabolism in patients with NAFLD compared with obese controls.

## Materials and Methods

### Human Subjects

Clinical studies were carried out on 16 patients recruited from the multidisciplinary NAFLD clinic at University Hospital Birmingham, with chronically elevated liver enzymes and evidence of hepatic steatosis on ultrasound. The diagnosis of NAFLD was made on histological analysis of clinically indicated biopsies after exclusion of other possible etiological factors (alcohol intake of >20 g/day, viral and autoimmune hepatitis and hepatototoxic drugs). 8 patients had hepatic steatosis and 8 had steatohepatitis. Renal function was normal and none were taking any drugs known to interfere with the HPA axis (glucocorticoids, anticonvulsants, estrogen treatment). Five patients had well controlled type 2 diabetes (2 steatosis patients on low dose metformin, 3 NASH patients – 2 on low dose metformin and one diet controlled). Patients on metformin had stopped medication for 2 days before participating in the study.

32 healthy obese control volunteers (BMI>30 kg/m^2^) were recruited by local advertisements. All had normal liver function biochemistry (AST, γGT, ALT, ALP and bilirubin).

Separate from the clinical study participants, liver samples were obtained from the liver tissue archive at the Centre for Liver Research, University of Birmingham and were used for *in vitro* gene and protein expression studies. These were all snap frozen samples that had been collected during the previous 24 month period, and stored at −80°C. All diagnoses were verified by histological analysis (NASH n = 5, normal transplant donor liver n = 5).

### Clinical Studies

Ethics Statement: The study and protocol received local ethics committee (Solihull research ethics committee) approval and written informed consent was obtained from all participants.

Patients were admitted to the research facility in the fasted state. Resting blood pressure (mmHg) and anthropometric measurements were taken (waist and hip circumference, BMI (kg/m^2^), and sagittal height (cm)). Venous blood samples were taken for fasting serum free fatty acids, liver function tests and other baseline biochemical blood measurements as per standard laboratory procedures.

Patients underwent body composition analysis using dual-energy X-ray absorptiometry (DXA) with a total body scanner (QDR 45OO; Hologic, Bedford, MA). Coefficients of variation (CVs) for multiple scans were <3%. Subcutaneous and visceral abdominal fat distribution was measured using a single 10 mm slice of computed tomography (CT) at the L3 vertebral level and analysed using commercial software (MeVis PULMO 3D 3.11, MeVIS Research GmbH, Bremen, Germany). A three dimensional analysis was carried out on the scan from which the fat area was calculated by dividing the volume results by the scan thickness. Total fat area and visceral fat area regions of interest (ROIs) were delineated by manually tracing a contour of each region. Fat pixels and therefore fat area were identified with threshold attenuation values between −50 to −250 hounsfield units as described previously [16]. The subcutaneous fat area was calculated by subtracting the visceral from total fat area. Data was expressed as 1) total, subcutaneous and visceral fat area, 2) the ratio of visceral to total fat (% visceral fat), 3) the ratio of visceral to subcutaneous fat (V∶S ratio). Patients also returned a 24 hour urine collection for steroid metabolite analysis.

On a second day of investigation, patients took 1 mg of dexamethasone orally at 2300h to suppress endogenous cortisol production, and attended the Clinical Research Facility at 0800h the following morning. After baseline 0900h measurements of cortisol and adrenocorticotropic hormone, a further 0.5 mg of dexamethasone and cortisone acetate (25 mg) were given orally. Serum cortisol concentrations were then measured at 20-min intervals for 240 min as previously reported [17].

### Biochemical analysis

Blood count, urea, creatinine and electrolytes, cholesterol, triglycerides, liver chemistry, and glucose were measured using standard laboratory methods (Roche Modular system; Roche, Lewes, U.K.). Plasma FFA were analysed on a COBAS BIO semiautomatic analyser (La Roche, Basel, Switzerland) using a NEFA-C Kit (Alpha Laboratories, UK).

Serum cortisol was assayed using a coat-a-count radioimmunoassay (Diagnostic Products, Los Angeles, CA) as per the manufacturer's guidelines. The ‘cortisol’ area under the curve generated following cortisone acetate administration was used as an index of hepatic 11β-HSD1 activity as previously described [17].

Urine samples were analyzed by gas chromatography/mass spectrometry, as reported previously [18], [19], measuring free and conjugated cortisol metabolites. The THF+5αTHF/THE, the cortols/cortolones, and the 11OH-androsterone+11OH-etiocholanolone/11-oxoetiocholanolone ratios represent acknowledged markers of global 11β-HSD1 activity, with a high ratio indicating increased 11β-HSD1 reductase activity, with the proviso that the urinary free F/E (UFF/UFE) ratio, reflecting 11β-HSD2 activity is normal. The 5αTHF/THF was used as a marker of 5α-reductase activity with a high ratio in the setting of increased absolute levels of urinary 5α-THF indicating increased activity.

### Real Time PCR

11β-HSD1, Glucocorticoid receptor α (GRα), and 5α-reductase 2 (SRD5A2) hepatic mRNA levels were measured by real-time PCR using an ABI 7500 system (Perkin-Elmer, Biosystems, Warrington, UK). PCR was performed in 25 µl reactions on 96-well plates. Reactions contained TaqMan universal PCR master mix (Applied Biosystems, Warrington, Cheshire, UK), 900 nmol primers, 100–200 nmol TaqMan probe and 25–50 ng cDNA. All reactions were multiplexed with primers specific for 18S rRNA (provided as a preoptimized mix; Perkin-Elmer, Beaconsfield, Bucks, UK) as an internal reference. All target gene probes were labelled with the fluorescent label FAM, and the 18S probe with the fluorescent label VIC. Reactions were as follows: 50°C for 2 min, 95°C for 10 min, and then 40 cycles of 95°C for 15 s and 60°C for 1 min. Data were analysed according to the manufacturer's guidelines and were obtained as Ct values (the cycle number at which logarithmic PCR plots cross a calculated threshold line) and used to determine dCt values (dCt = Ct of the target gene minus Ct of the internal reference, 18S). Probes and primers for all genes were provided by ‘assay on demand’ (Applied Biosystems). Arbitrary units were used with the transformation [AU = 1000*2^−dCt^] to express results obtained.

### Immunohistochemistry and Immunofluorescence

Five micron thick acetone fixed frozen liver sections with severe NASH and normal donor livers were cut onto coated glass slides. The slides were treated with methanol-hydrogen peroxide 0.1% to block endogenous peroxidase activity for 20 minutes. After washing in phosphate buffered saline (PBS) sections were incubated in 20% normal donkey serum for 30 minutes and then with polyclonal antibody to 11β-HSD1 [20] at a dilution of 1 in 100 in 10% donkey serum for 45 minutes. Secondary antibody, donkey antisheep IgG peroxidase conjugate (1∶200), was added to sections for 30 min. Slides were developed using 3,3′-diaminobenzidine and were counterstained with Mayer's hematoxylin. Immunofluorescence was carried out to detect co-localisation of 11β-HSD1 with the CD68 macrophage marker (purified mouse anti human CD68, BD Pharmingen). 11β-HSD1 primary antibodies were as described above. Alexa Fluor 488 donkey anti sheep IgG and Alexa Flour 546 rabbit anti mouse IgG were used at a dilution of 1∶100 and slides were covered in foil for the remainder of the procedure. Slides were mounted in VectaShield hard set mounting medium with DAPI (Vector Labs).

### Preparation of Liver Microsomes

Human liver microsomes were prepared from 4 human normal livers and 5 livers with NASH livers by differential centrifugation techniques as described previously [21]. Microsomal fractions were resuspended in a buffer containing 20 mm NaCl, 1 mm MgCl_2_, 100 mm KCl, 20 mm Mops, pH 7.2, and were snap-frozen under liquid nitrogen. Microsomal protein concentration was determined using the Bio-Rad protein assay with bovine serum albumin as a standard as per the manufacturer's instructions (Bio-Rad). The integrity of the microsomal membranes was assessed by using the mannose-6-phosphatase assay [22], which showed a latency greater than 95% in all preparations.

### Immunoblotting

SDS-PAGE was performed by the method of Laemmli [23] with 10 µg of liver microsomal protein on 11% acrylamide minigels using a Bio-Rad Mini-PROTEAN II apparatus (Bio-Rad). Following electrophoresis, proteins were transferred to Immobilon-P membrane (Millipore Corp., Bedford, MA). Nonspecific protein binding was blocked by incubating membranes in 20% nonfat milk, 0.1% Tween 20 in phosphate-buffered saline at 25°C for 1 h. Membranes were then incubated with an in-house raised polyclonal antibody to human 11β-HSD1 at a dilution of 1∶1000 for 12 h at 4°C. Following 3×10-min washes in phosphate-buffered saline, 0.1% Tween 20, membranes were incubated with secondary antibody (goat anti-sheep IgG peroxidase-conjugate) at a dilution of 1∶25,000 for 1 h at room temperature. Bound peroxidase-conjugated IgG was visualized using ECL detection kit (Amersham Biosciences, Buckinghamshire, UK) by exposing membranes to x-ray film (Kodak, France). Membranes were reprobed with anti-beta Actin antibody [mAbcam 8226] (HRP) as a loading Control at 1∶20,000.

### Statistical Analysis

Data are presented as means ± SE unless otherwise stated. Area under the curve (AUC) analysis was performed using the trapezoidal method. For comparison of single variables between control, steatosis and steatohepatitis groups, one way analysis of variance (ANOVA) was used to identify variables with differences between groups and *t* tests were used (Mann Whitney test was used where data were not normally distributed). Analysis was performed using SPSS Statistics 17.0 software.

## Results

### Clinical and biochemical characteristics of participants

We characterised the metabolic phenotype and hepatic cortisol metabolism in patients with histologically proven NAFLD compared to healthy obese control. Compared with the control group, waist∶hip ratios and sagittal height were significantly higher in the NASH group. While % visceral fat was also highest in the NASH group this did not achieve significance. Liver transaminases were similar in both steatosis and NASH group and significantly higher than controls. Insulin resistance using the HOMA-IR model was highest in the NASH group. Baseline clinical and biochemical characteristics of both groups are presented in Table 1.

**Table 1:** Baseline clinical characteristics of patients with hepatic steatosis, NASH and controls.

Variable	Control subjects	Steatosis	NASH
*n*	32	8	8
age (years)	47±2.0	37±3.0^f^	54±2.0
BMI kg/m^2^	32.4±0.9	37.1±3.1	36.5±1.8
waist∶hip ratio	0.9±0.01^d^	0.96±0.1	1.0±0.03
sag height (cm)	23.3±0.6^d^	22±1^f^	27.9±1.2
%Fat∼	37.4±1.2^a^	29.7±2.8	36.1±2.5
% visceral fat	37.6±5.4	43.3±4.4	51.4±4.8
V∶S ratio	0.6±0.1	0.83±0.2	1.17±0.2
Creat (µmol/L)	89±3	96±4	89±4
Total cholesterol (mmol/L)	6.8±1.4	5.3±0.7	5.2±0.4
HDL cholesterol (mmol/L)	1.4±0.1	1.1±0.1	1.3±0.1
Triglycerides (mmol/L)	1.3±0.1^b^ ^c^	2.5±0.4	2.1±0.4
ALT (iu/L)	24±2^b^ ^d^	93±23	62±13
ALP (u/L)	166±7	170±12	204±30
AST (iU/L)	22±0.9^b^ ^d^	40±6	51±8
γGT (iu/L)	22±2^b^ ^d^	68±21	119±48
fasting FFA (µmol/L)	328±17	431±64	413±36
fasting glucose (mmol/L)	4.7±0.1^d^	4.8±0.2^e^	6.5±0.7
Systolic blood pressure (mmHg)	134±4	133±2	135±5
Diastolic blood pressure (mmHg)	76±3	77±5	79±3
Cortisol post dex suppression (nmol/L)	26±5	12±3	26±5
Fasting insulin (mU/L)	9.7±1.8^d^	16±5.1	27±7.7
HOMA-IR	2.21±0.5^d^	2.6±1.0	7.4±2.6

Data are presented as means ± SE.

∼ Whole body fat measured by DXA. CT measured visceral and subcutaneous fat.

a controls vs steatosis p<0.05,

b controls vs steatosis p<0.01, controls vs NASH p<0.05,

d controls vs NASH p<0.01,

e steatosis vs NASH p<0.05,

f steatosis vs NASH p<0.01. (Dex: dexamethasone).

### Urinary steroid metabolite analysis

**Figure 1:**
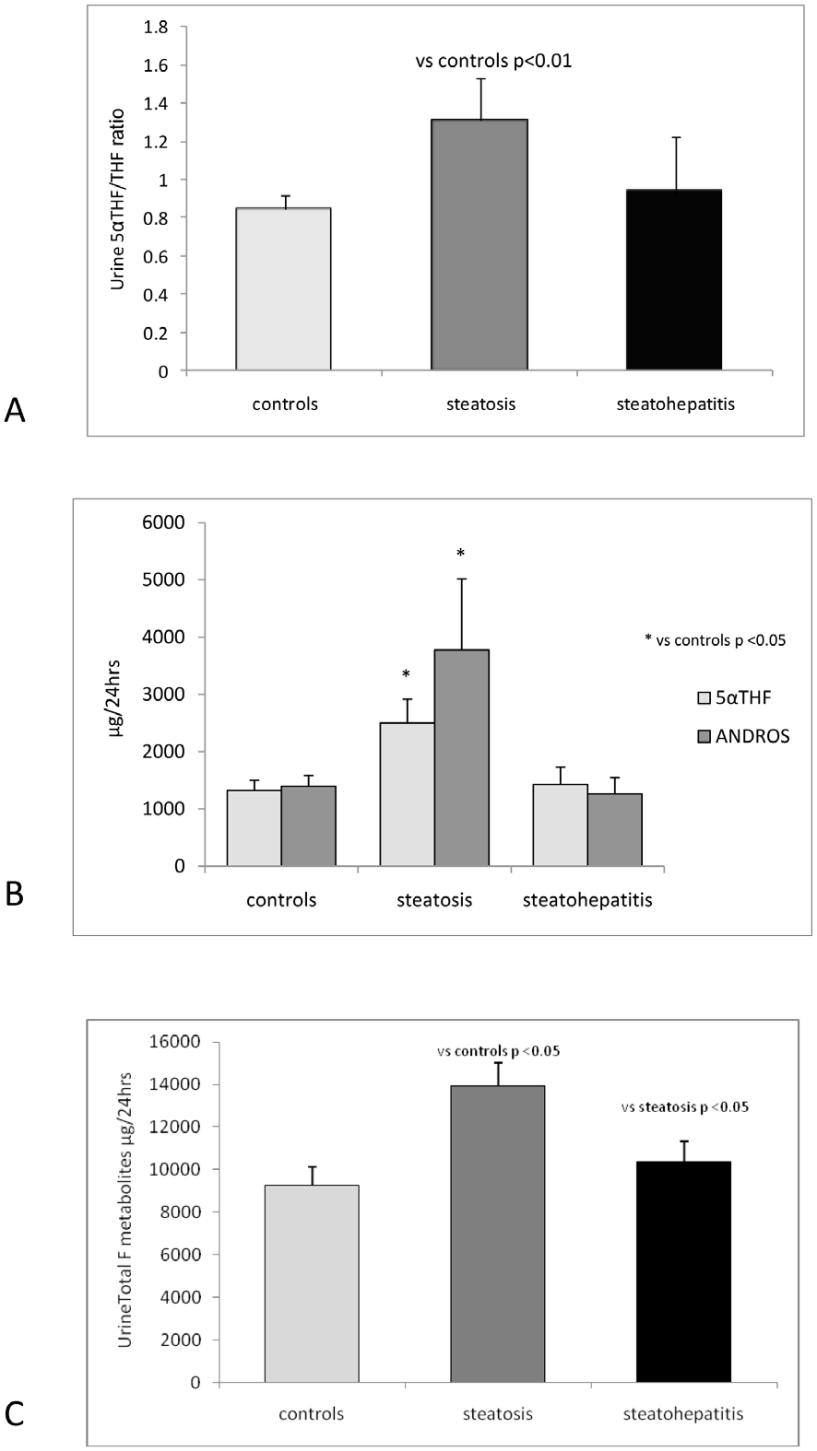
24 hour urine steroid metabolite analysis from patients with steatosis and steatohepatitis compared with obese controls. (A): 5α-reductase activity as depicted by the urinary 5αTHF/THF ratio (mean ± SEM). (B): total 24 Urine 5α-reduced metabolites (mean ± SEM) (Andros: androsterone). (C): total 24 hr Urine F metabolites (mean ± SEM).

24 h urinary steroid metabolite analysis by GC/MS demonstrated increased cortisol clearance with higher 5αR (reflected by urine 5αTHF/THF and An/Et ratios) in patients with hepatic steatosis only, Figure 1A. 5α-, and not 5β-reduced metabolites were increased in the steatosis group, Figure 1B. Absolute values are presented in Table 2.

**Table 2:** Urinary steroid metabolites and ratios in patients with steatosis, NASH and control patients.

Steroid (µg/24 h)	controls	steatosis	NASH
*n*	32	8	8
Cortisol	71±7	78±8	112±20^a^
Cortisone	120±11	127±20	162±39
THE	3426±346	5626±554	3447±326^c^
THF	1624±169	1973±199	1759±231
5α-THF	1324±178	2500±417^a^	1429±300
a-cortol	316±27	379±42	496±90^a^
β-cortol	457±36	533±102	515±97
α-cortolone	1303±111	1899±206^a^	1789±256
β-cortolone	621±57	834±112	641±91
Total F metabolites	9266±857	13949±1075^a^	10351±984^d^
**Ratios**			
F/E	0.61±0.03	0.65±0.06	0.74±012
(THF+5αTHF)/THE	0.89±0.04	0.81±0.06	0.96±0.13
cortols/cortolones	0.43±0.02	0.33±0.02^a^	0.42±0.04
5αTHF/THF	0.84±0.07	1.31±0.22^a^	0.93±0.3
An/ET	1.11±0.10	1.99±0.31^b^	1.55±0.6

Mean absolute values are shown (µg/24 h) +/− SEM.

a P<0.05 vs controls,

b P<0.01 vs controls,

c P<0.05 vs steatosis,

d P<0.01 vs steatosis. (An: Androsterone, Et: Etiochoanolone, THE: tetrahydrocostione, THF: tetrahydrocortisol).

**Figure 2:**
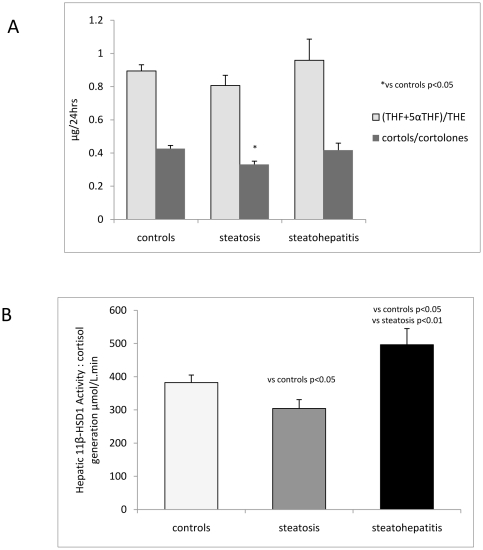
11β-HSD1 activity assessed by: (A) 24 hr urine cortols/cortolones and 5αTHF+THF/THE ratios (mean ± SEM) in patients with steatosis and steatohepatitis compared with controls. (B) Hepatic cortisol generation measured by cortisol generation profiles (mean AUC ± SEM) in patients with steatosis and steatohepatitis compared with controls.

In addition, total urine cortisol metabolites were significantly increased in patients with steatosis consistent with increased glucocorticoid production rate, Table 2, Figure 1C. The urinary THF+5αTHF/THE ratio was lower in the steatosis group and elevated in the steatohepatitis group but this did not reach statistical significance. However the cortols/cortolones ratio, which also reflects 11β-HSD1 activity, was significantly reduced in the steatosis group, Table 2, Figure 2A. Importantly the urine UFF/UFE ratio was similar between groups indicating that there was no difference in extrahepatic 11β-HSD2 activity, Table 2.

### Cortisol Generation Profiles

Endorsing the urinary steroid metabolite data, cortisol generation from oral cortisone was decreased in patients with steatosis compared with controls. In contrast, patients with steatohepatitis had significantly increased cortisol generation consistent with increased hepatic 11β-HSD 1 activity compared with controls and patients with steatosis, Figure 2B.

### 11β-HSD1 expression studies

**Figure 3:**
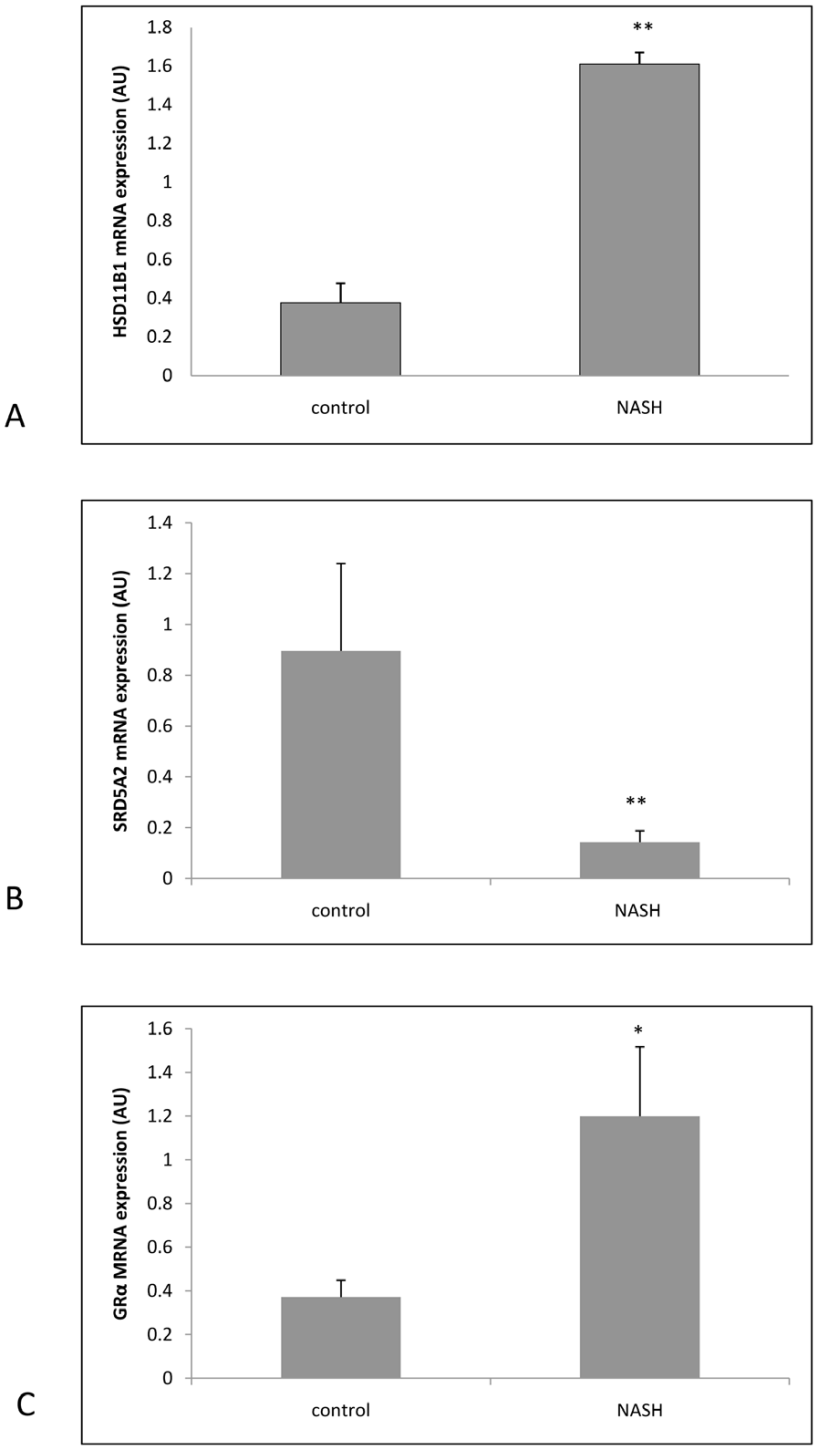
Real time PCR mRNA expression data on whole liver samples from 5 normal patients and 5 NASH patients (expressed as arbitrary units ± SEM) for (A)HSD11B1 (11β-HSD 1), (B)SRD5A2 (5α-reductase 2), (C)GRα. ** p<0.01 NASH vs controls; * p<0.05 NASH vs controls.

Supporting the above data, 11β-HSD1 mRNA expression in explant livers with NASH was significantly higher compared with normal controls (dCT NASH 9.65±0.29 vs 11.96±0.29, p<0.01 NASH vs control), Figure 3A. SRD5A2 mRNA expression was significantly decreased in NASH (dCT NASH 13.3±0.01 vs 10±0.01, p<0.01 NASH vs control), Figure 3B and GRα mRNA expression was significantly increased in NASH (dCT NASH 10.4±0.3 vs 11.7±0.3, p<0.05 NASH vs control), Figure 3C).

**Figure 4:**
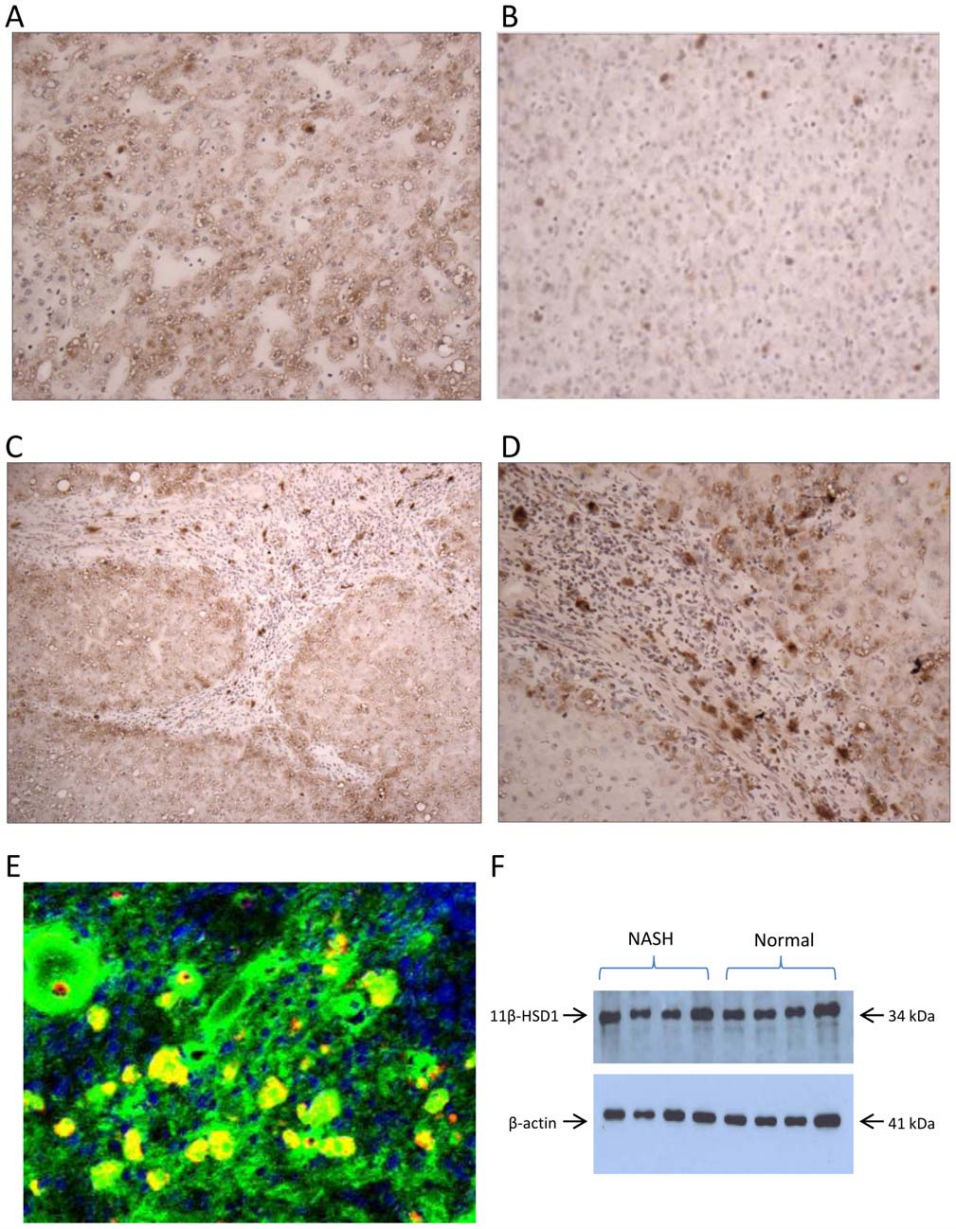
Hepatic 11β-HSD 1 immunoreactivity in patients with severe NASH compared to normal controls. There was generally increased staining for 11β-HSD1 throughout the liver parenchyma in (A) NASH samples compared with (B) Normal liver ×20. (C) and (D) Increased staining at the limiting plate in peri-septal areas and strongly staining specific cells within the inflammatory infiltrate in NASH ×10(C) and ×20(D) (E) Confocal microscopy on severe NASH cryosections. Green - 11β-HSD1, red – CD68 IgG macrophage marker, yellow – colocalisation of 11β-HSD1 and CD68 positive macrophages. (F) Western blot analysis of human liver microsomes from normal and NASH livers.

These results were further supported at the protein level by immunohistochemistry; protein expression for 11β-HSD1was increased in NASH livers compared with normals. Specifically, intense staining was seen in hepatocytes in periseptal areas. There was also intense staining in inflammatory cells within cirrhotic nodules with morphology in keeping with macrophages. Immunofluorescence studies confirmed these cells as CD68 positive cells co-localising with 11β-HSD1 Figure 4 A–E. Immunoblotting for 11β-HSD1 of microsomal preparations of livers from patients with NASH compared with normals did not show any significant difference in expression, Figure 4F. This represents a discrepancy between protein immunoblotting and mRNA expression in the same samples. However the histological appearance of the NASH samples provides some clues to the possible explanation for this. When comparing protein expression by immunoblotting per µg of liver microsomal protein, the overall 11β-HSD1 protein expression may be similar between normal and diseased groups because the immunoblotting technique does not acknowledge localized changes in expression.

## Discussion

**Figure 5:**
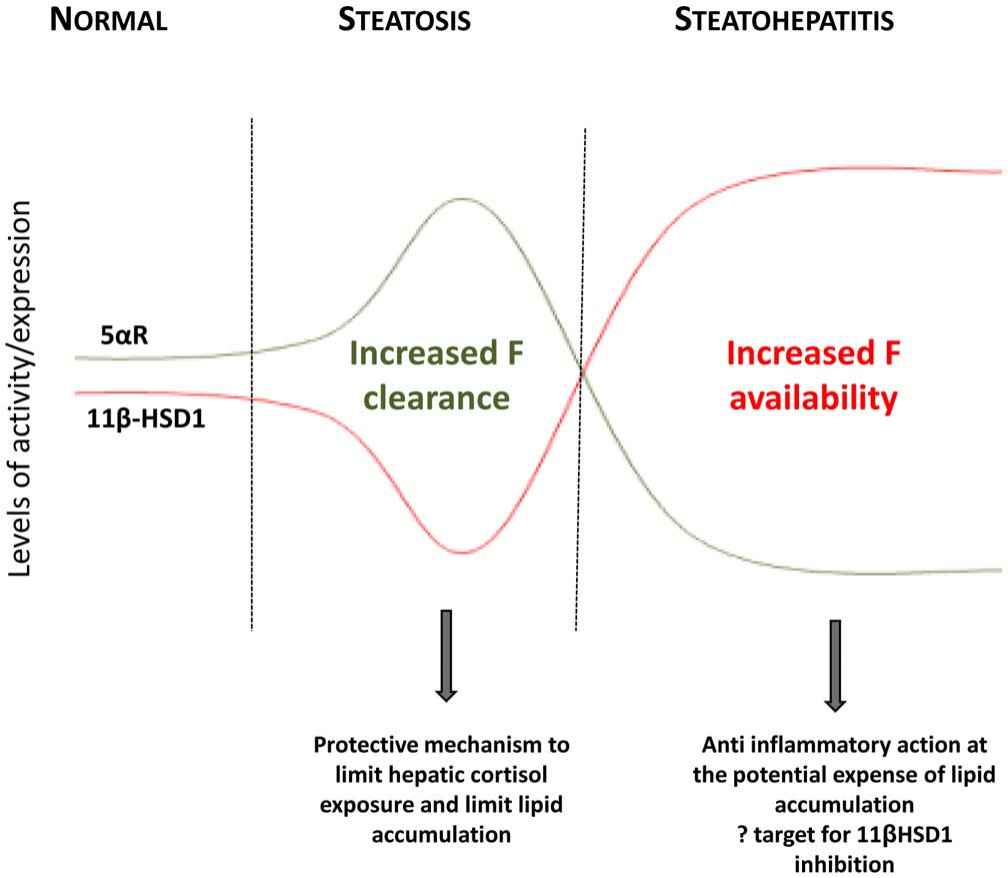
Schematic: Hepatic glucocorticoid metabolism and its modulation in response to disease progression in NAFLD.

We have defined hepatic glucocorticoid metabolism in patients with the full spectrum of NAFLD. In the early stages of NAFLD, characterized by hepatic steatosis alone, hepatic cortisol clearance predominates, driven by increased 5αR activity, and decreased cortisol generation from 11β-HSD1 with a consequent activation of the HPA axis and adrenal glucocorticoid production. With disease progression and worsening inflammation and liver injury, there is induction of hepatic 11β-HSD1 expression and activity that increase hepatic glucocorticoid levels, with hepatic glucocorticoid exposure maximized by increased expression of GRα and decreased expression and activity of 5α-reductase. In steatohepatitis 11β-HSD1 expression is specifically intense in CD68 positive macrophages, and may imply a role in response to chronic inflammation. Collectively these results provide a key insight into the pathophysiology of the NAFLD disease spectrum, with a switch from inactivation to activation of hepatic glucocorticoid levels as patients move from steatosis to NASH, Figure 5.

The first suggestion of a role of 11β-HSD1 in NAFLD came with the observation that transgenic mice over expressing 11β-HSD1 in adipose tissue develop the full phenotype of the metabolic syndrome including hepatic steatosis. Conversely recombinant mice with global deletion of 11β-HSD1 are protected from many of these features including hepatic steatosis [24], [25]. Transgenic mice overexpressing the 11β-HSD1 gene selectively in the liver under the transcriptional control of the human apoE gene, exhibit fatty liver (but not steatohepatitis) with increased hepatic triglyceride accumulation and impaired hepatic lipid clearance [26]. Furthermore, selective 11β-HSD1 inhibition reduced hepatic triglyceride concentration by nearly 30% and increased in vivo hepatic fat oxidation and expression of related genes in rats fed an obesogenic diet [27].

We postulate that the increase in hepatic cortisol clearance by 5α-reductases, and decreased 11β-HSD1 driven hepatic cortisol generation in hepatic steatosis is a protective mechanism to preserve hepatic metabolic phenotype by limiting hepatic cortisol exposure and glucocorticoid induced deleterious effects. These include ongoing hepatic lipogenesis, and gluconeogenesis with increased glucose output which worsen hepatic and peripheral insulin resistance. Indeed, recent work from our group has shown a reduction in 5α-reductase activity with weight loss in otherwise healthy obese subjects [28]. Women with polycystic ovarian syndrome who are characterized by a susceptibility to the metabolic syndrome also have increased 5α-reductase activity and adrenocortical drive [29]. Both these groups of patients have an increased propensity to develop NAFLD. Our data support previously published findings of a subtle activation of the HPA axis in patients with NAFLD, [13], [14]. This may be secondary to the increased clearance of hepatic glucocorticoid by the 5α-reductase enzymes or reduction in11β-HSD1as shown in our steatosis patients. Supportive *in-vitro* data with gene and protein expression studies from steatotic human liver samples would be a valuable extension for this work as such tissue becomes available.

In contrast, there is a clear drive to increase hepatic glucocorticoid availability in steatohepatitis due to altered regulation at multiple levels. These include reduced A ring reductase mediated glucocorticoid clearance, increased glucocorticoid receptor expression and increased 11β-HSD1 activity and expression. 11β-HSD1 protein expression in NASH is increased throughout the liver compared with controls. Increased GRα expression (which is expressed homogenously throughout the liver parenchyma in all hepatocytes without any specific zonal distribution, [30]) would further maximize immediate effects of increased hepatic glucocorticoid production. BMI data of individuals from which the donor liver (normal control) samples were obtained were not known, and it is possible that the BMI of this group was lower than the obese control group used in the clinical study. However, our previous work has shown that in simple obesity, there is a reduction in the generation of serum cortisol from dexamethasone-suppressed values after the administration of oral cortisone reflecting decreased hepatic 11β-HSD1 activity [9]. Hence it may be expected that hepatic 11β-HSD1 gene expression in simple obesity is also reduced compared with non obese normal livers. Furthermore, in NASH livers 11β-HSD1 expression was specifically increased in hepatocytes in periseptal areas and in CD68 positive macrophages within inflamed cirrhotic septa. 11β-HSD1 expression was most intense in CD68 positive macrophages, even more than in hepatocytes; collectively these results would indicate a specific role for 11β-HSD1 glucocorticoid production in the inflammatory process that occurs in NASH. Previous studies have shown macrophage expression of 11β-HSD1 in the context of distinct disease models of acute inflammation notably murine studies where macrophage 11β-HSD1 activity rapidly increases during the development of acute peritonitis [31]. Our human study in NASH has shown for the first time that macrophage 11β-HSD1 expression is specifically intense in a chronic inflammatory process. These results clearly lead to a number of exciting possibilities with respect to the role of 11β-HSD1 in steatohepatitis. With relevance to the inflammatory response it would be important to discern the phenotype of the response of these macrophages. Previous studies have shown that macrophage 11β-HSD1 expression is stimulated by IL-4 and IL-3 cytokines, both examples of Th2 cytokines that promote anti inflammatory responses. In the peritonitis model referred to above, macrophage 11β-HSD1 expression was important in the induction of phagocytosis of apoptotic neutrophils [31]. However, treatment of a murine macrophage cell line with 11β-HSD1 inhibitors was able to reduce the proinflammatory cytokine response following lipopolysaccharide treatment [32]. Macrophage 11β-HSD1 mediated glucocorticoid production may therefore be a central mechanism to fine tune the phenotype of the inflammatory response. This may be to limit injury in chronic inflammation, and promote pro resolution mechanisms particularly in acute inflammation. The role of the differential activation of Type 1 (pro-inflammatory), and Type 2 (anti-inflammatory) macrophages in determining the outcome of liver inflammation has only recently been appreciated [33]. Increased hepatocyte 11β-HSD1 expression, particularly in periseptal areas would further directly expose inflamed septa to glucocorticoids. However overall increased hepatic glucocorticoid would also promote hepatic lipogenesis and hence be expected to worsen hepatic steatosis. Studies on other tissues, including synovium from patients with rheumatoid arthritis [34], human and rodent colitis [35], aortic smooth muscle cells [36], and granulosa cells in the inflammatory response to ovulation [37], all show a consistent picture of induction of cell specific 11β-HSD1 gene expression in response to pro inflammatory cytokines, TNFα and IL-1β being the most commonly implicated [38]. The molecular mechanism by which 11β-HSD1 is induced in response to cytokines is not entirely clear, but key transcription factors of the C/EBP family play a crucial role [39], [40].

Simple hepatic steatosis is a relatively benign entity in the NAFLD disease spectrum with only a 2% risk of developing progressive disease in a twenty year period. However, the presence of fibrosis or inflammation at diagnosis is associated with a risk of developing NASH cirrhosis of up to 50% in a two year period [1]. The factors implicated in the crucial switch between simple steatosis and NASH are not entirely clear. Increased liver fat is pivotal to inflammation in NAFLD, and thus the increased supply of free fatty acids to the liver, associated with adipose tissue insulin resistance and obesity is a key factor in the development of hepatic inflammation in NAFLD. Our data show increased fasting serum FFA in patients with NAFLD compared with controls although this not achieve significance as in previously described studies. Adipose tissue insulin resistance may occur in obesity in part through the infiltration of macrophages which release pro inflammatory cytokines such as TNFα, IL-6 and IL1β [41]. Once FFA are taken up by the liver, as well as being oxidized and stored as triglyceride, they activate the transcription factor NFκB, a key regulator of gene transcription of proinflammatory cytokines, adhesion molecules, and chemokines [42]. What results is a cycle of hepatic injury and inflammation. The cytokines released from hepatocytes, in particular TNFα activate classic inflammatory cells, as well as Kupffer cells which generate more cytokines, further contributing to hepatic oxidative stress by promoting FFA oxidation, which enhances the hepatic injury that occurs by cytokine driven hepatocyte apoptosis and necrosis [43].

The histological appearance of NASH and alcoholic steatohepatitis are similar. Fatty change is also commonly seen in hepatitis C infection and in some cases is associated with steatohepatitis. Our in vivo studies showed increased hepatic glucocorticoid generation in patients with alcoholic liver disease [44] suggesting that 11β-HSD1 may be increased in steatohepatitis regardless of the underlying cause. Longitudinal studies investigating the role of hepatic 11β-HSD1 in disease progression and outcome of hepatic steatosis would provide valuable data.

This work has defined hepatic glucocorticoid metabolism in progressive NAFLD, which can be summarized into two distinct phases of altered regulation of hepatic cortisol metabolism; increased hepatic cortisol clearance in steatosis, and increased hepatic cortisol regeneration in NASH. Failure to regulate in this way may worsen the phenotype of liver disease i.e. drive hepatic steatosis or unchecked progressive hepatic inflammation. This is an exciting area of investigation that clearly warrants further study but may impact upon the role of selective 11β-HSD1 inhibitors in the treatment of patients with the Metabolic Syndrome. 11β-HSD1 inhibition may be favorable in treating hepatic steatosis by limiting hepatic lipid deposition, but paradoxically may worsen an inflammatory response in the presence of NASH. Hence the therapeutic benefit of 11β-HSD1 inhibition may critically depend on the histological stage of NAFLD.
